# Estimation of the potential GDP by a new robust filter method

**DOI:** 10.1007/s10100-023-00851-7

**Published:** 2023-03-28

**Authors:** Éva Gyurkovics, Tibor Takács

**Affiliations:** 1 Mathematical Institute, Budapest University of Technology and Economics, Műegyetem rkp. 3, Budapest, 1521 Hungary; 2 Corvinus University of Budapest, 8 Fővám tér, Budapest, 1093 Hungary

**Keywords:** Potential GDP, Robust filtering, Polytopic and quadratically bounded uncertainties, Linear matrix inequality, Unobserved components model, Trend-cycle decomposition, C13, C22, C32, C52

## Abstract

The first purpose of this paper is to propose a theoretically new robust filter method to estimate non-observable macroeconomic indicators. The second purpose is to apply the proposed method to estimate the Hungarian potential GDP in 2000–2021. The novelty of the proposed filter method is that — unlike papers published so far — it does not require the stability of the dynamic model, only a partial stability condition must be satisfied. Moreover, such time-dependent uncertainties and nonlinearities can arise in the model that satisfy a general quadratic constraint. An important advantage of the proposed robust filter method over the traditional Kalman filter is that no stochastic assumptions is needed that may not be valid for the problem at hand. The proposed filter method has never been applied to estimate the potential GDP. To estimate the Hungarian potential GDP, the proposed method is applied using uni-, bi- and trivariate models. Estimations up to 2021 has not been published yet for the Hungarian economy. The examined period includes both the financial world crisis and the Covid-19 crisis. The results of the different models are consistent. It turned out that the economic policy was very procyclical after 2012, and the GDP gap was still positive during and also after the Covid-19 crisis.

## Introduction

The concept of potential GDP (Gross Domestic Product) is of great importance for both economic analysts and economic policy makers. It refers to the maximum value added of the national economy that is sustainable in the sense that it does not accelerate inflation. If current GDP is persistently above potential, the economy needs to be cooled; if it is below potential, it needs to be stimulated according to the principles of counter-cyclical policy. Since the potential GDP is not a measurable indicator, it can only be estimated based on some kind of model calculation. The determination of the potential GDP requires a trend-cycle decomposition, where the trend is the potential GDP. In practice, different models are used, but they raise different methodological problems, and their application may lead to significantly different results.

Each model can be grouped in different ways (see e.g. Alpysbaeva et al. (2021), Apokin et al. (2016), Bhoi et al. (2017), Brand et al. (2021), Cerra et al. (2000), EU (2019), Pauna et al. (2021), Sallam et al. (2019), St-Amant et al. (1997)). Models can be distinguished according to whether they include only GDP itself as a variable or whether they also include other variables that are theoretically relevant to the evolution of potential GDP. The other variables are usually unemployment and inflation, which are used to interpret the sustainable evolution of the GDP. Univariate models usually use some form of trending or decomposition method, the most common in practice being the standard Hodrick-Prescott filter (see e.g. Bhoi et al. (2017), Mohr (2005), IMF (2014), Harvey et al. (1993), St-Amant et al. (1997)). The advantage of this is that it is technically easy to use, but its applicability is greatly reduced by the fact that the estimate becomes uncertain at the end of the period under consideration, whereas the method should provide a guide to the potential level for economic policy as it approaches the present. Multivariate models rely on the relationships between variables: typically unemployment and inflation are represented in the models to interpret sustainability (maximum output with non-accelerating price and wage inflation). They are therefore also called structured models. A typical form of structured models is based on macro-level production functions (e.g. Cerra et al. (2000), Konuki (2010)). A major drawback of this method is that it requires a choice of different types of production functions and it is difficult to provide reliable data for parameter estimation. Filter models are often used to calculate potential GDP (e.g. Cuche et al. (1999), Enders et al. (2015), Guillén et al. (2014), Konuki (2010), St-Amant et al. (1997)). Here, the Kalman filter, well known from engineering applications, is applied to a stochastic dynamical system given in a state-space representation. This provides a way to estimate the unobservable variables of the dynamical equations in such a way that the observable output variable of the system is matched as closely as possible to the actual measured data. However, to apply the Kalman filter, we need to make certain stochastic assumptions that are not necessarily satisfied.

*The contribution of this paper is twofold:*

(a) a theoretically new robust filter method is developed to determine the potential GDP, which is exempt from stochastic assumptions,

(b) the proposed method is applied to determine the Hungarian GDP.

*The paper is organized as follows.* Section 2 is the problem statement. Section 3 presents new robust models to estimate the potential GDP. The new theoretic results are presented in Sect. 4. Section 5 discusses the results of the application of the models, while Sect. 6 draws the conclusions.

*Notations:* Standard notations are used. In particular, $A^T$ and $A^{-1}$ denote the transpose and the inverse of matrix *A*. $A > 0$ ($A<0$) means that the matrix *A* is symmetric and positive (negative) definite. $I_n$ denotes the $n\times n$ identity matrix, $0_{n\times m}$ denotes the $n\times m$ zero matrix. The symbol $*$ denotes a symmetric structure in the matrix. Norms of discrete-time functions $\{f(t): t \in {\textbf{N}}\rightarrow {\textbf{R}}^p\}$ are defined as $\left\| f \right\| _{\infty }^{2} = \sup _{t\in {\textbf{N}}} \left\{ f(t)^Tf(t)\right\} ,$ $\left\| f\right\| _{2}^2= \sum _{t=0}^{\infty } f(t)^Tf(t).$ Function *f* is of class $\ell _2$, if $\left\| f\right\| _{2}< \infty .$

## Problem statement

In the literature, various types of filter models are used to estimate directly unobservable variables like the potential GDP. Some authors tend to present their models as universal models, often applying the same model for different economies, although the consideration of specificities is essential both for the choice of the model type and for the calibration of the parameters.

When choosing the model, different aspects should be taken into consideration. The inclusion of the unemployment and/or the inflation raises the problem that the different variables are measured on different scales, and this is hardly discussed in the literature. The applied models are sometimes presented as universal ones that can be applied for different economies using practically the same coefficients. The sign of the coefficients can be expected the same based on practice and theory, but the magnitude obviously depends on the chosen scales. In the dynamic equation of the unemployment, the coefficient of the cyclical component of the GDP is often interpreted as the Okun-parameter (see e.g. Evans (2018), Melolinna et al. (2019)) and it is calibrated around $-$0.5 as the Okun’s law suggests. It is sometimes used then for cross-country comparisons, although the other variable in the dynamic equation, i.e. the GDP is measured on a different scale, and this latter scale (currency and unit) can vary by country. This means that the multivariate dynamic models used for filtering cannot be universal, the coefficients should be calibrated for each country; there is no universal Okun-parameter. Moreover, practice has also proven that the Okun’s law is far from being universal, it has never been derived from any theory; the relationship between the potential GDP and the unemployment may be different across economies of similar model and development level. Similar problem emerges, if the inflation is included in the dynamic model. It is suitable to use multivariate models for filtering, if it is possible to give a reliable estimation of these coefficients by some external method for the examined period. One of the advantages of the proposed robust filtering is that, at least in a restricted way, it is suitable to treat such estimation uncertainties, i.e. to take into consideration the imperfect knowledge of the dynamics.

The estimation of the potential GDP by filtering is done for a past period, but the most important result for economic policy is whether the output gap is positive or negative at the end of the period under consideration, i.e. in the present. The most commonly used trend calculation method for estimating potential GDP in practice is the Hodrick-Prescott (HP) filter. Unfortunately, this method gives uncertain results just at the end of the period, as the last point will be overweighted in determining the trend. Another advantage of robust filtering, on the other hand, is that the estimation error converges to zero as time progresses, so that more reliable results are obtained at the end of the filtering period. In robust filtering, depending on the choice of the starting point of the filter, the first few periods may have larger deviations.

Different methods can be used to estimate the parameters of the dynamic equations. Some authors apply the maximum-likelihood approach with Monte-Carlo simulation, see e.g. Arouba et al. (2013), Basistha (2007), Bulligan et al. (2019), Busetti et al. (2016), González-Astudillo et al. (2021), Guillén et al. (2014), IMF (2014), Kuttner (1994), Melolinna et al. (2019), Morley et al. (2003). Another applicable approach is the use of structural equations treated by the least squares method, see e.g. Brand et al. (2021), Lee (2019), Margirier (2018), Sallam et al. (2019). AR/VAR models are applied in e.g. Alpysbaeva et al. (2021), Apokin et al. (2016), Perron et al. (2009), St-Amant et al. (1997). The cycle parameters can be based on spectral analysis, see Harvey et al. (1993). The parameters can be based also on expert opinions, see e.g. Konuki (2010), Malecek et al. (2021), which are not necessarily based on model calculations. Any of these methods can be used to estimate the model parameters, but the result is always subject to uncertainty. If the different methods provide only certain intervals for the parameters, the energy-to-peak filter for the uncertain system can be applied, which is free from any stochastic considerations, and can treat the uncertainties of different nature.

The filtering models for estimating potential GDP frequently apply the Kalman filter (see e.g. Alpysbaeva et al. (2021), Apokin et al. (2016), Arouba et al. (2013), Basistha (2007), Cerra et al. (2000), Clark (1987), Cuche et al. (1999), Guillén et al. (2014), Konuki (2010), Kuttner (1994), Malecek et al. (2021), Melolinna et al. (2019), Us (2018)), i.e. the dynamic equations are considered to be stochastic, the error terms are assumed to be normally distributed with known covariance matrices. When the Kalman-filter is applied, each of the model parameters is supposed to follow normal distribution with zero mean and known variance matrix. Considering the nature of the problem, the assumption of a symmetric distribution may not be a well-based assumption. The basic idea of the robust approach is that uncertainties can be taken into consideration without any stochastic assumptions. The uncertainties stemming from the imperfect knowledge of system dynamics can be modelled by nonlinear deterministic functions, for which only certain boundedness assumptions are made. In this case, the specific forms of the nonlinear uncertainties are not supposed to be known, only the class of functions is specified. The exogenous disturbances affecting the dynamics are also modelled by deterministic functions of class $l_2$. Two possible approaches of modelling uncertainties could be applied here. If the linear parameter-varying (LPV) models are used, the set of systems parameters may be anywhere in a polytope. Another usual approach is to define the set of admissible uncertainties by certain bounding conditions. The new model proposed here is of this latter type, providing a flexible way to capture uncertainties avoiding the possible numerical problems of the LPV systems, which may not be tractable numerically, if the constraining polytope has too many vertices. The method proposed by this paper, employs the special structure of dynamics of the problem. We consider all nonlinearities and uncertainties of the model together, and assume that they are quadratically bounded.

The focus of this paper is to use measured data i.e. the output of a given dynamical system to estimate unobservable variables: the potential GDP, the natural rate of unemployment and the reference level of inflation for a past period. To solve this problem, we propose a new robust filter method, where both nonlinear uncertainties and exogenous disturbances are taken in consideration in the systems dynamics. The first contribution of this paper is to propose a new robust energy-to-peak filter method, which is suitable to estimate non-observable economic indicators such as the potential GDP. The method proposed to estimate the potential GDP is itself theoretically new, since it does not assume the stability of the nominal (i.e. the uncertainty-free) system, which is a usual assumption in previously published papers (with the only exception of Gyurkovics et al. 2022 (cf. the references therein)). Moreover, the nonlinear terms that appear in the model are not assumed to be known, as e.g. in de Souza (2019). The second contribution is the estimation of the Hungarian potential GDP for the Hungarian economy. The examined period includes both the financial crisis in 2008–2012 and the Covid-19 crisis in 2020–2021. Such calculations have not been published yet.

## Models to estimate the potential GDP

We propose here a class of models to estimate the potential GDP by filtering. With appropriate specification, these dynamic systems may have nonlinear terms and non-identified parametric uncertainties, which may depend on time.

All these models are based on the multiplicative relation $Y_t=Y_{p,t} Y_{c,t}$, where *t* denotes the time, $Y_t$ is the observed GDP, $Y_{p,t}$ is the directly non-observable trend component, i.e. the potential GDP, and $Y_{c,t}$ is the cyclical component. Taking the logarithm of both sides, the basic identity $y(t)=y_p (t)+y_c (t)$ is obtained. Applying this transformation we consider the following trivariate model.

1 $$\begin{aligned} y_p (t+1)&=y_p (t)+g(t)+w^{(y_p ) } (t), \end{aligned}$$

2 $$\begin{aligned} g(t+1)&=g(t)+w^{(g) } (t), \end{aligned}$$

3 $$\begin{aligned} \begin{bmatrix} y_c(t+1) \\ y_c^*(t+1) \end{bmatrix}&= \varrho \begin{bmatrix} \cos { \omega } &{} -\sin { \omega } \\ \sin { \omega } &{} \cos { \omega } \end{bmatrix} \begin{bmatrix} y_c(t) \\ y_c^*(t) \end{bmatrix} + \begin{bmatrix} w_{\varrho }^{(y_c )}(t) \\ w_{\varrho }^{(y_c^* )}(t) \end{bmatrix}, \end{aligned}$$

4 $$\begin{aligned} u_p (t+1)&= u_p (t)+w^{(u_p ) } (t), \end{aligned}$$

5 $$\begin{aligned} u_c (t+1)&= u_p (t)+\gamma _u y_c (t)+ \varphi _u \left( t,y_c\right) +w^{(u_c ) } (t), \end{aligned}$$

6 $$\begin{aligned} \pi _p (t+1)&= \pi _p (t)+w^{(\pi _p ) } (t), \end{aligned}$$

7 $$\begin{aligned} \pi _c (t+1)&= \pi _p (t)+\gamma _\pi y_c (t)+ \varphi _{\pi } \left( t,y_c\right) +w^{(\pi _c ) } (t), \end{aligned}$$

8 $$\begin{aligned} y(t)&= y_p (t)+y_c (t), \end{aligned}$$

9 $$\begin{aligned} u(t)&= u_p (t)+u_c (t), \end{aligned}$$

10 $$\begin{aligned} \pi (t)&= \pi _p (t)+\pi _c (t), \end{aligned}$$

where *g*(.) is the logarithm of the growth rate of the potential GDP, $$y_c^*(.)$$ is an auxiliary variable to generate the cycle, $$u_p (.)$$ is the natural rate of unemployment, $$u_c (.)$$ is a cyclical component, $$\pi _p (.)$$ is the natural or reference rate of inflation, $$\pi _c (.)$$ is a cyclical component, while *u*(.) and $$\pi (.)$$ are the unemployment rate and the inflation, respectively. The terms $$w^{(.) } $$ are the uncertain inputs that are assumed to be $$\ell _2$$ functions in this and later models. The numerical parameters $$\varrho ,$$ $$\omega ,$$ (or $$a_1=\varrho \cos { \omega } $$ and $$a_2 =\varrho \sin { \omega } $$), $$\gamma _u,$$ $$\gamma _\pi $$ are not supposed to be known exactly, but some sets will be specified to which they belong. The nonlinear functions $$\varphi _u \left( t,y_c\right) $$ and $$\varphi _{\pi } \left( t,y_c\right) $$ represent the unidentified uncertainties.

Equations (1)–(7) describe the dynamics of the process, while (8)–(10) represent the measured output.

Specifically, Eqs. (1)–(3) describe the dynamics of the two components of the GDP, namely of the trend and of the cyclical component. The trend component is identified as the potential GDP. It increases more or less at the same rate in every time period, see (1)–(2). The cyclical component is captured by trigonometric terms, where $0< \omega < \pi$ is the cycle frequency, and $0<\rho <1$ is the damping parameter, see (3).

In Eqs. (4)–(5), the dynamics of the unemployment rate is modelled. To include the unemployment in the dynamic model is a natural idea, since the potential GDP is a level of value added that is sustainable in that sense that it does not accelerate the inflation. Theory suggests that there is a level of unemployment that does not accelerate price and wage dynamics (the non-accelerating inflation rate of unemployment—NAIRU, or the non-accelerating wage rate of unemployment—NAWRU). The dynamic equations describing the relationship between unemployment and potential GDP are based on (the often debated) various forms of Okun’s law, which theoretically shows how much GDP is below its potential level if unemployment is 1% above its natural rate. By Okun’s law we may assume that parameter $\gamma _u$ is negative, but its value cannot be derived directly from any form of the Okun’s law. Given the qualitative nature of this law, it is reasonable to assume that $u_p$ depends on $y_p$ nonlinearly, but the exact way of this dependence is unknown. This is represented by the uncertain nonlinear function $\varphi _u \left( t,y_c\right)$ satisfying certain growth conditions specified below.

Equations (6)–(7) describe the dynamics of the inflation rate. Given the definition of potential GDP, it is logical to include inflation as a separate variable in the model. For this type of model, authors mostly refer to the Phillips curve, although the theoretical problems with this have been discussed for decades. Following Melolinna et al. (2019), we present inflation in a similar way to unemployment. This means that we also interpret the natural or reference rate of inflation, which we assume to be a low inflation rate that does not distort economic processes, but is still far enough away from the deflationary danger zone. In the dynamic equation of the inflation, the cyclical component of GDP is also the explanatory variable, but here the coefficient is theoretically positive: in theory, an increase is associated with an increase in the inflation rate, while a fall is associated with its decrease. This means that the modelling of inflation is formally analogous to that of the unemployment, but parameter $\gamma _\pi$ is supposed to be positive. Its value has to be calibrated, as well. Similarly to the modelling of the unemployment, it is reasonable to allow an uncertain nonlinear dependence on $y_p$ described by $\varphi _{\pi } \left( t,y_c\right) ,$ which also satisfies certain growth conditions defined below.

Papers Apokin et al. (2016), Bhoi et al. (2017), Brand et al. (2021) Cerra et al. (2000), González-Astudillo et al. (2021), IMF (2014), Melolinna et al. (2019), St-Amant et al. (1997), Us (2018) apply similar, but more specific models assuming fully linear models with given constant numerical parameters.

### Remark 1

As mentioned in Harvey (1985), representation (3) can be rewritten also as an AR(2) process

$$\begin{aligned} y_c (t+1)=\phi _1 y_c (t)+ \phi _2 y_c (t-1) + \varepsilon ^c_y \end{aligned}$$

with $\phi _1 =2\rho cos\omega$ and $\phi _2= -\rho ^2$. Though the two representations are equivalent, we apply here the explicit model of the cyclical process, although the proposed method could be applied to the AR(2) form, too.

In what follows, we shall investigate models with different complexity that will be referred to as Model 1, Model 2 and Model 3 as follows.

*Model 1* is univariate model including only the GDP. It is given by Eqs. (1)–(3) and (8). Papers Alqarelleh (2019), Bulligan et al. (2019), Harvey et al. (2003), Mohr (2005) considered the more specific case with constant $\varrho$ and $\omega$.

*Model 2* is bivariate, which includes only two variables: the GDP and the unemployment rate. This means that this model includes Eqs. (1)–(5) and (8)–(9). Papers Evans (2018), González-Astudillo et al. (2021), Margirier (2018) investigated this model with constant parameters $\varrho ,$ $\omega ,$ $\gamma _u,$ and with $\varphi _u \left( t,y_c\right) \equiv 0$ and $\varphi _{\pi } \left( t,y_c\right) \equiv 0$.

*Model 3* is the trivariate model given by Eqs. (1)–(10).

### Assumption 1

The numerical parameters of the models that may be time-independent or time varying, have known bounds, while the uncertain nonlinearities satisfy the condition

11 $$\begin{aligned} \varphi _{u} \left( t,y_c\right) \left( \varphi _{u} \left( t,y_c\right) -\theta _u y_c \right) \le 0, \varphi _{\pi } \left( t,y_c\right) \left( \varphi _{\pi } \left( t,y_c\right) -\theta _{\pi } y_c \right) \le 0, \end{aligned}$$

where $\theta _u$ and $\theta _{\pi }$ are given numbers.

The uncertain nonlinearity satisfying (11) is referred to as *Lur’e-type uncertainty*.

## Robust energy-to-peak filtering

### General problem formulation

In this paper two types of uncertain discrete-time systems will be considered:

12 $$\begin{aligned} {\mathcal {S}}_1: x(t+1)&=A_x(\alpha ) x(t)+ B_x(\alpha ) w(t), \end{aligned}$$

13 $$\begin{aligned} y(t)&=C_y(\alpha ) x(t) + B_{y}(\alpha ) w(t), \end{aligned}$$

14 $$\begin{aligned} z(t)&= L(\alpha )x(t), \end{aligned}$$

and

15 $$\begin{aligned} {\mathcal {S}}_2: x(t+1)&=A_x x(t)+ H_{x}p_x(t)+B_x w(t), q_x(t) = A_{q} x(t)+ G_x p_x(t), \end{aligned}$$

16 $$\begin{aligned} y(t)&=C_y x(t) + H_{y}p_y(t)+B_{y} w(t), q_y(t) = C_{q} x(t)+ G_y p_y(t), \end{aligned}$$

17 $$\begin{aligned} z(t)&= Lx(t). \end{aligned}$$

In both systems, $x\in {\textbf{R}}^{n_{x}}$ is the state, $w\in {\textbf{R}}^{n_{w}}$ is the exogenous disturbance, which belongs to $\ell _2$, $y \in {\textbf{R}}^{n_{y}}$ is the measured output, $z(t)\in {\textbf{R}}^{n_z}$ is the signal to be estimated, and all matrices are of appropriate dimension.

System ${\mathcal {S}}_1$ represents a polytopic uncertain system, having coefficient matrices that are assumed to belong to the polytope

18 $$\begin{aligned} \Pi&= \left\{ A_x(\alpha ), B_x(\alpha ),C_y(\alpha ), B_y(\alpha ), L(\alpha )\right\} = \sum _{i=1}^{\sigma }\alpha _i \left\{ A^{(i)}_x,B^{(i)}_x,C^{(i)}_y,B^{(i)}_y,L^{(i)} \right\} \end{aligned}$$

where $\alpha \in \Sigma =\left\{ \alpha : \ \sum _{i=1}^{\sigma }\alpha _i =1, \; \alpha _i \ge 0\right\}$, and $\sigma$ is the number of vertices of the polytope. The current values of the parameters are not assumed to be available for computations.

In ${\mathcal {S}}_2,$ the system matrices are assumed to be known, and all systems nonlinearities/uncertainties are represented by the uncertain input functions $p_x,$ $p_y,$ possibly depending on *t*, *x*,  and by the the uncertain output functions $q_x,$ $q_y.$ Denote $$p = \begin{bmatrix} p_x^T&p_y^T \end{bmatrix}^T \in {\textbf{R}}^{l_p},$$ and $$q = \begin{bmatrix} q_x^T&q_y^T \end{bmatrix}^T \in {\textbf{R}}^{l_q}.$$ According to the different kinds of uncertainties/nonlinearities that may arise, *p* and *q* are divided into $s \ge 1$ subcomponents $p=(p_{1}^{T},\ldots ,p_{s}^{T})^{T},$ $q=(q_{1}^{T},\ldots ,q_{s}^{T})^{T}$ and matrix $G = \text{ diag } \left\{ G_x, \ G_y \right\}$ assumed to have a corresponding block-diagonal structure $G = \text{ diag } \left\{ G_{1},\ldots ,G_{s} \right\}$. The only available information about *p* and *q* is that their values are constrained by the set19where $l_p=l_{p1}+\cdots + l_{ps}$, $l_q=l_{q1}+\cdots + l_{qs}$. For $i=1,\ldots ,s$, $Q_{0i}=Q_{0i}^{T}$, $R_{0i}=R_{0i}^{T} \ge 0$ and $S_{0i}$ are constant matrices of appropriate dimension. The set (19) will be called the set of admissible nonlinearities/uncertainties. It has to be pointed out that — in contrast to de Souza (2019) — the nonlinearities that may occur in (15)–(16) are not supposed to be known.

Set $$Q_0 = \text{ diag }\left\{ Q_{01},\ldots ,Q_{0s}\right\} ,$$ $$R_0 = $$ $$\text{ diag }\left\{ R_{01},\ldots ,R_{0\,s}\right\} , $$ $$S_0 = \text{ diag }\big \{S_{01},\ldots ,S_{0\,s}\big \}.$$ The following condition will be required for $$\Omega $$.

#### Assumption 2

        $$R_0 \ge 0,$$         $$Q_{0}+G^{T}S_{0}^{T} +S_{0} G + G^{T}R_{0} G<0.$$

Observe that Assumption 2 assures the well-posedness of system (15)–(16), and that the origin is an equilibrium point of the unperturbed uncertain/nonlinear system.

It is worth noting that the considered model of uncertainties involves several types of uncertainties frequently investigated in the literature. For example, by appropriate choice of $Q_{0}$, $S_{0}$ and $R_{0}$, one can describe norm bounded and Lur’e type uncertainties of the previous section, as well as linear fractional, generalized positive real and sector bounded uncertainties.

*The aim* is to find a filter ${\mathcal {F}}$

$$\begin{aligned} {\mathcal {F}}: \quad {\hat{x}}(t+1)&= {\hat{A}} {\hat{x}}(t) + {\hat{H}}y (t), \\ {\hat{z}}(t)&= {\hat{J}} {\hat{x}}(t) + {\hat{K}}y(t), \end{aligned}$$

where ${\hat{x}}(t)\in {\textbf{R}}^{n_{x}},$ ${\hat{z}}(t)\in {\textbf{R}}^{n_z}$ so that the following conditions hold for any $\alpha \in \Sigma$:

1. The error $e(t)=z(t)-{\hat{z}}(t)$ satisfies condition $\lim _{t \rightarrow \infty }e(t)=0,$ if $w(t)\equiv 0.$

2. A prescribed disturbance attenuation level $\gamma$ is guaranted for all nonzero $w(t) \in \ell _2$, i.e. for zero initial value

20 $$\begin{aligned} \left\| e \right\| _{{\infty }}^{2} < \gamma ^2 \left\| w\right\| _{{2}}^2. \end{aligned}$$

Then filter ${\mathcal {F}}$ is said to be a *filter with a guaranteed robust energy-to-peak performance bound* $\gamma .$

The robust filter design methods proposed in the literature (with the only exception of Gyurkovics et al. (2022)) can only be applied, if the magnitude of the eigenvalues of matrix $A_x$ is strictly less then 1,  which is not satisfied for the models of Sect, 2. Similarly to the paper Gyurkovics et al. (2022), it will be shown, how the requirement of asymptotic stability of the unperturbed system can be relaxed in order to apply the robust filtering to estimate the potential GDP.

#### Condition 1

(Relaxed stability condition.) There are $$n_{x_1} $$ variables which are not affected by uncertainties, i.e. with possible renaming of variables, *x* can be partitioned as $$x^T=\begin{bmatrix}x_1^T, x_2^T \end{bmatrix},$$ $$(x_1 \in {\textbf{R}}^{n_{x_1}}, \ x_2 \in {\textbf{R}}^{n_{x_2}} )$$ and the coefficient matrices can be written as

$$\begin{aligned} A_x=&\begin{bmatrix} A_{11} &{} A_{12} \\ 0 &{} A_{22} \end{bmatrix} , \qquad C_y=\begin{bmatrix}C_1&C_2 \end{bmatrix},\qquad L=\begin{bmatrix}L_1&L_2 \end{bmatrix}, \\ H_x=&\begin{bmatrix} H_{x_1} \\ H_{x_2} \end{bmatrix} , \qquad \qquad A_q=\begin{bmatrix} 0&A_{q2} \end{bmatrix} , \qquad C_q=\begin{bmatrix} 0&C_{q2} \end{bmatrix} , \end{aligned}$$

where matrices $A_{12},$ $A_{22},$ $C_{2}$ and $L_{2}$ may depend on the parameter $\alpha ,$ if system ${\mathcal {S}}_1$ is considered. Moreover, the magnitude of the eigenvalues of $A_{22}$ is less than 1.

Considering the analogous partition of the filter data, the matrices of the filter are chosen to satisfy

21 $$\begin{aligned} {\hat{A}}_{11}=A_{11}-{\hat{H}}_1C_1, {\hat{A}}_{21}=-{\hat{H}}_2C_1 \text{ and } {\hat{J}}_1=L_1-{\hat{K}}C_1. \end{aligned}$$

### Robust filtering result for the polytopic system ${\mathcal {S}}_1$

In order to formulate the design conditions, define a matrix $\Phi (m,n)\in {\textbf{R}}^{n\times m}$: $\Phi (m,n)= I_{n}$, if $n=m$,

$$\begin{aligned} \Phi (m,n)=\begin{bmatrix} I_{n} \\ 0 \end{bmatrix}, \text { if } n<m, \Phi (m,n)= \begin{bmatrix} I_{m}&0 \end{bmatrix}, \text { if } n>m. \end{aligned}$$

#### Theorem 1

Consider system ${\mathcal {S}}_1,$ and suppose that Condition 1 is satisfied. Let the data of the filter ${\mathcal {F}}$ satisfy (21). If for given scalar parameters $\beta ,$ $c_1,$ $b_j,$ $d_j,$ ($j=1,\ldots ,6$), there exist matrices $P_i=P_i^T, \, R_i, \, S_i, \, U,\, V,\, X \in {\textbf{R}}^{n_{cl}\times n_{cl}},$ $W_i, \, Y \in {\textbf{R}}^{n_w \times n_{cl}},$ $(i=1,\, \ldots , \,\sigma ),$ $F_1 \in$ ${\textbf{R}}^{n_{x_1}\times n_{x_1}},$ $F_2 \in {\textbf{R}}^{n_{x_2}\times n_{x_2}},$ ${\overline{A}}_{12} \in {\textbf{R}}^{n_{x_1}\times n_{x_2}},$ ${\overline{A}}_{22} \in {\textbf{R}}^{n_{x_2}\times n_{x_2}},$ ${\overline{H}}_1 \in {\textbf{R}}^{n_{x_1}\times n_y},$ ${\overline{H}}_2 \in {\textbf{R}}^{n_{x_2}\times n_y},$ ${\hat{K}} \in {\textbf{R}}^{n_z \times n_y}$ ${\hat{J}}_2 \in {\textbf{R}}^{n_z \times n_{x_2}}$ such that the following linear matrix inequalities (LMIs) admit a solution:

22 $$\begin{aligned} \begin{bmatrix} -P_i &{} *&{} *\\ 0 &{} - I &{} *\\ {\mathcal {L}}^{(i)} &{} {\mathcal {D}}^{(i)}&{} -\gamma ^2 I \end{bmatrix}&< 0, i=1,\ldots ,\sigma , \end{aligned}$$

23 $$\begin{aligned} \begin{bmatrix} \Omega ^{(i)}+\Upsilon \Gamma _{2}^{(i)}+{\Gamma _{2}^{(i)}}^T\Upsilon ^T &{} *\\ \beta \Xi ^T + \Gamma _{2}^{(i)} &{} -\beta \left( F+F^T\right) \end{bmatrix}&<0, i=1,\ldots ,\sigma , \end{aligned}$$

wherethen ${\mathcal {F}}$ is a *filter with a guaranteed robust energy-to-peak performance bound* $\gamma$. If (22) and (23) are feasible, then the filter matrices can be obtained by

24 $$\begin{aligned} {\hat{A}}_{12}=F_1^{-1}{\overline{A}}_{12}, {\hat{A}}_{22}=F_2^{-1}{\overline{A}}_{22}, {\hat{H}}_1=F_1^{-1}{\overline{H}}_{1}, {\hat{H}}_2=F_2^{-1}{\overline{H}}_{2}, \end{aligned}$$

and (21).

Proof. The proof is given in Gyurkovics et al. (2022).

### Robust filtering result for the uncertain/nonlinear system ${\mathcal {S}}_2$

Consider now system ${\mathcal {S}}_2$ with filter ${\mathcal {F}}.$ For the variable $${\bar{x}}=\begin{bmatrix} x^T&{\hat{x}}^T \end{bmatrix} ^T$$ one can write the closed-loop system as

25 $$\begin{aligned} {\bar{x}}(t+1)&={\bar{A}}_{{\bar{x}}} {\bar{x}}(t)+ {\bar{H}}_{{\bar{x}}}p(t)+{\bar{B}} w(t), \end{aligned}$$

26 $$\begin{aligned} q_{{\bar{x}}}(t)&= {\bar{A}}_{{\hat{q}}} {\bar{x}}(t)+ G p(t), \end{aligned}$$

27 $$\begin{aligned} {\bar{z}}(t)&= z(t)-{\hat{z}}(t)= \begin{bmatrix} L&{\hat{J}} \end{bmatrix} {\bar{x}}(t), \end{aligned}$$

where

$$\begin{aligned} {\bar{A}}=&\begin{bmatrix} A &{} 0 \\ {\hat{H}}C_y &{} {\hat{A}} \end{bmatrix} , {\bar{H}}=\begin{bmatrix} H_{x } &{} 0 \\ 0 &{} {\hat{H}} H_{y} \end{bmatrix} , {\bar{B}}=\begin{bmatrix} B_x \\ {\hat{H}} B_y \end{bmatrix} , {\bar{A}}_q=\begin{bmatrix} A_{q} &{} 0 \\ C_{q} &{} 0 \end{bmatrix} . \end{aligned}$$

Let us introduce a new system of coordinates by the definition

$$\begin{aligned} \xi = \begin{bmatrix} (x_1-{\hat{x}}_1)^T&(x_2 -{\hat{x}}_2)^T&{\hat{x}}_2^T&( x_1+{\hat{x}}_1)^T \end{bmatrix}^T, \end{aligned}$$

and take the partition $$\xi =\left[ \xi _1^T \; \xi _2^T \right] ^T,$$ $$\xi _1 \in {\textbf{R}}^{n_{cl}},$$ $$\xi _2 \in {\textbf{R}}^{n_{x_1}} $$ ($$ n_{cl}=n_{x_1}+2n_{x_2}$$). Performing the corresponding transformation on (25)–(27), one obtains the error system $${\mathcal {E}}$$ as follows:282930where

$$\begin{aligned} {\mathcal {A}}&= \begin{bmatrix} A_{11}-{\hat{H}}_1C_1 \; \; &{} A_{12}-{\hat{H}}_1C_2\; \; &{} A_{12}-{\hat{H}}_1 C_2-{\hat{A}}_{12} \\ -{\hat{H}}_2 C_1 &{} A_{22}-{\hat{H}}_2 C_2 &{} A_{22}-{\hat{H}}_2 C_2- {\hat{A}}_{22}\\ {\hat{H}}_2 C_1 &{} {\hat{H}}_2 C_2 &{} {\hat{A}}_{22}+{\hat{H}}_2 C_2 \end{bmatrix}, \\ {\mathcal {A}}_{21}&= \begin{bmatrix} {\hat{H}}_{1} C_1& A_{12} +{\hat{H}}_{1}C_2& A_{12} +{\hat{H}}_{1}C_2+{\hat{A}}_{12} \end{bmatrix} , {\mathcal {A}}_{22}= A_{11}, \\ {\mathcal {H}}&= \begin{bmatrix} H_{x_1} &{} -{\hat{H}}_1H_y \\ H_{x_2} &{} -{\hat{H}}_2 H_y \\ 0 &{} {\hat{H}}_2B_y \end{bmatrix}, {\mathcal {B}}= \begin{bmatrix} B_{x_1}-{\hat{H}}_1B_y \\ B_{x_2}-{\hat{H}}_2 B_y \\ {\hat{H}}_2B_y \end{bmatrix}, {\mathcal {A}}_q = \begin{bmatrix} 0 &{} A_{q2} &{} A_{q2} \\ 0 &{} C_{q2} &{} C_{q2} \end{bmatrix},\\ {\mathcal {H}}_2&= \begin{bmatrix} H_{x_1}& {\hat{H}}_1 H_y \; \end{bmatrix}, {\mathcal {B}}_2= \begin{bmatrix} B_{x_1}+{\hat{H}}_1B_y \end{bmatrix}, \\ {\mathcal {L}}&= \begin{bmatrix} L_1-{\hat{K}}C_1&L_2-{\hat{K}}C_2&L_2-{\hat{K}}C_2-{\hat{J}}_2 \end{bmatrix}, {\mathcal {D}} ={{-}}{\hat{K}} B_y. \\ \end{aligned}$$

One can see that the subspace ${\mathcal {H}}=\left\{ \xi \in {\textbf{R}}^{2n_x}: \xi _1=0 \right\}$ is invariant for ${\mathcal {E}},$ if $w(t)\equiv 0,$ and $e(t)\rightarrow 0$ as $t\rightarrow \infty ,$ if the distance $d(\xi (t),{\mathcal {H}})\rightarrow 0$ as $t\rightarrow \infty .$ Therefore, it is sufficient to investigate the behavior of the error system ${\mathcal {E}}$ with respect to this subspace.

Before formulating the filter design conditions for system ${\mathcal {E}}$, some further notations are needed. For any positive numbers $\varepsilon _i,$ $\tau _i,$ $i=1, \ldots , s,$ let

$$\begin{aligned} \underline{\tau }&= \text{ diag } \{ \tau _1 I_{l_{p1}} ,\ldots , \tau _s I_{l_{ps}} \}, \; \underline{\underline{\tau }} = \text{ diag } \{ \tau _1 I_{l_{q1}} ,\ldots , \tau _s I_{l_{qs}} \}, \\ \underline{\varepsilon }&= \text{ diag } \{ \varepsilon _1 I_{l_{p1}} ,\ldots , \varepsilon _s I_{l_{ps}} \}, \; \underline{\underline{\varepsilon }}=\text{ diag } \{ \varepsilon _1 I_{l_{q1}} ,\ldots , \varepsilon _s I_{l_{qs}} \}. \end{aligned}$$

#### Theorem 2

Consider system ${\mathcal {S}}_2,$ and suppose that Assumption 2 and Condition 1 are satisfied. Let the data of the filter ${\mathcal {F}}$ satisfy (21). If for given scalar parameters $\beta ,$ $c_i$, ($i=1,2,3$), $b_j,$ $d_j,$ ($j=1,\ldots ,6$), there exist matrices $P=P^T, \, R_i, \, S_i, \, Z_i, \,U_i,\, V_i,$ ($i=1,2$), $X_j, \, Y_j,$ ($j=1,\ldots ,7$), $F_1,\, F_2,$ ${\overline{A}}_{12},$ ${\overline{A}}_{22},$ ${\overline{H}}_1, \, {\overline{H}}_2,$ ${\hat{K}}, \, {\hat{J}}_2$ and positive numbers $\varepsilon _i,$ $\tau _i, ($ $i=1, \ldots , s$), such that the following linear matrix inequalities (LMIs) admit a solution:

31 $$\begin{aligned} \begin{bmatrix} -P+ {\mathcal {A}}_q^T \underline{\underline{\varepsilon }} R_0 {\mathcal {A}}_q &{} *&{} *&{} *\\ 0 &{} - I &{} *&{} *\\ \left( {\underline{\varepsilon }} S_0 + G^T \underline{\underline{\varepsilon }} R_0 \right) {\mathcal {A}}_q &{} 0 &{} \underline{\varepsilon } Q_{0}+G^{T}S_{0}^{T}\underline{\varepsilon } +\underline{\varepsilon }S_{0} G + G^{T}\underline{\underline{\varepsilon }}R_{0} G &{} *\\ {\mathcal {L}} &{} {\mathcal {D}}&{} {\mathcal {H}} &{} -\gamma ^2 I \end{bmatrix}&< 0, \end{aligned}$$

32 $$\begin{aligned} \begin{bmatrix} \Lambda +\Delta \Gamma _{1}+ {\Gamma _{1}}^T \Delta ^T -\Upsilon \Gamma _{2}-{\Gamma _{2}}^T\Upsilon ^T &{} * \\ \beta (\Delta {\mathcal {I}} + \Upsilon F)^T + \Gamma _{2} &{} -\beta \left( F+F^T\right) \end{bmatrix}&<0, \end{aligned}$$

where3334then ${\mathcal {F}}$ is a *filter with a guaranteed robust energy-to-peak performance bound* $\gamma$ for system ${\mathcal {S}}_2.$ If (31) and (32) are feasible, then the filter matrices can be obtained by (21) and (24).

#### Proof

The proof follows a similar line, as it frequently can be seen in the literature, one has only to use a Lyapunov function defined in a way usual at stability of sets. Indeed, let $P \in {\textbf{R}}^{n_{cl}\times n_{cl}}$ be positive definite, and define *V* by $V(\xi )= \xi ^T \left[ I_{n_{cl}} 0\right] ^T P \left[ I_{n_{cl}} 0\right] \xi .$ Then there are positive numbers $\mu _1, \, \mu _2$ such that $\mu _1 \Vert \xi _1\Vert ^2 \le V(\xi )\le \mu _2\Vert \xi _1\Vert ^2.$ On the other hand,

35 $$\begin{aligned}&V(\xi (t+1))-V(\xi (t))-w(t)^Tw(t) \nonumber \\& = \begin{bmatrix} \xi _1(t) \\ w(t) \\ p(t) \end{bmatrix}^T \begin{bmatrix} I &{} 0 &{} 0 \\ 0 &{} I &{} 0 \\ {\mathcal {A}} &{} {\mathcal {B}} &{} {\mathcal {H}} \end{bmatrix}^T \begin{bmatrix} - P &{} 0 &{} 0\\ 0 &{} -I &{} 0 \\ 0 &{} 0 &{} P \end{bmatrix} \begin{bmatrix} I &{} 0 &{} 0 \\ 0 &{} I &{} 0 \\ {\mathcal {A}} &{} {\mathcal {B}} &{} {\mathcal {H}} \end{bmatrix} \begin{bmatrix} \xi _1(t) \\ w(t)\\ p(t) \end{bmatrix}. \end{aligned}$$

$$\square $$

If $(p^T,q^T)^T \in \Omega ,$ then for any positive numbers $\tau _i,$ ($i=1,\ldots ,s$),

36 $$\begin{aligned} 0&\le (*) (*)\begin{bmatrix} \underline{\tau }Q_0 &{} \underline{\tau }S_0 \\ *&{} \underline{\underline{\tau }}R_0 \end{bmatrix} \begin{bmatrix} 0 &{} 0 &{} I\\ {\mathcal {A}}_q &{} 0 &{} G \end{bmatrix} \begin{bmatrix} \xi _1(t) \\ w(t)\\ p(t) \end{bmatrix} . \end{aligned}$$

Adding (36) to (35), and introducing the notation

37 $$\begin{aligned} \Psi= & {} (*) \begin{bmatrix} -P &{} 0 &{} 0 &{} 0 &{} 0 \\ 0 &{} -I &{} 0 &{} 0 &{} 0 \\ 0 &{} 0 &{} P &{} 0 &{} 0 \\ 0 &{} 0 &{} 0 &{} \underline{\tau }Q_0 &{} \underline{\tau }S_0 \\ 0 &{} 0 &{} 0 &{} S_0^T \underline{\tau } &{} \underline{\underline{\tau }}R_0 \end{bmatrix} \begin{bmatrix} I &{} 0 &{} 0\\ 0 &{} I &{} 0 \\ {\mathcal {A}} &{} {\mathcal {B}} &{} {\mathcal {H}}\\ 0 &{} 0 &{} I\\ {\mathcal {A}}_q &{} 0 &{} G \end{bmatrix}, \end{aligned}$$

one can immediately see that

38 $$\begin{aligned} V(\xi (t+1))-V(\xi (t))-w(t)^Tw(t) \le \begin{bmatrix} \xi _1(t) \\ w(t)\\ p(t) \end{bmatrix} ^T \Psi \begin{bmatrix} \xi _1(t) \\ w(t)\\ p(t) \end{bmatrix}, \end{aligned}$$

therefore, if $\Psi <0,$ then the left hand side of (38) is less than $-\mu _3 \left( \Vert \xi _1(t) \Vert ^2+ \Vert w(t) \Vert ^2 \right. \left. + \Vert p(t) \Vert ^2\right)$ with some $\mu _3>0.$ This implies that, for any admissible uncertainty, $\Vert \xi _1(t) \Vert \rightarrow 0$ and $\Vert e(t) \Vert \rightarrow 0$ when $t\rightarrow \infty ,$ if $w(t)\equiv 0.$ By summing up, it follows from (38) and $\Psi <0$ that, for $\xi _1(0)=0,$

39 $$\begin{aligned} V(\xi (t)) < \sum _{k=0}^{t-1} w(k)^T w(k). \end{aligned}$$

By using Schur complement to inequality (31), applying congruence with $$\begin{bmatrix} \xi _1(t)^T&w(t)^T&p(t)^T \end{bmatrix}^T,$$ and observing that inequality (36) remains valid, if $\tau _i$-s are replaced by arbitrary positive $\varepsilon _i$-s, one can verify that

40 $$\begin{aligned} e(t)^Te(t) \le&\gamma ^2\left( V(\xi (t))+ w(t)^Tw(t)\right) . \end{aligned}$$

Now, (39) and (40) immediately imply (20).

In what follows, we prove that inequality (32) implies $\Psi <0.$

Inequality $\Psi <0$ can equivalently be written as

41 $$\begin{aligned} { \Gamma _{\bot }^{0}}^T \Lambda ^{0} \Gamma _{\bot }^{0} <0,& \text{ with } \nonumber \\ \Lambda ^{0}= \begin{bmatrix} P &{} *&{} *&{} *&{} *\\ 0 &{} -P &{} *&{} *&{} *\\ 0 &{} 0 &{} -I &{} *&{} *\\ 0 &{} 0 &{} 0 &{} \underline{\tau }Q_0 &{} *\\ 0 &{} 0 &{} 0 &{} S_0^T \underline{\tau }&{} \underline{\underline{\tau }}R_0 \end{bmatrix},& \Gamma _{\bot }^{0} = \begin{bmatrix} {\mathcal {A}} &{} {\mathcal {B}} &{} {\mathcal {H}}\\ I &{} 0 &{} 0\\ 0 &{} I &{} 0 \\ 0 &{} 0 &{} I\\ {\mathcal {A}}_q &{} 0 &{} G \end{bmatrix}. \end{aligned}$$

The application of Finsler’s lemma (see e.g. in Appendix) gives that (41) is equivalent to the existence of a matrix $\Delta ^0$ such that

42 $$\begin{aligned} \Omega ^0 \doteq&\Lambda ^{0} + \Delta ^0 \Gamma ^0 + { \Gamma ^0}^T {\Delta ^0} < 0, \end{aligned}$$

where

$$\begin{aligned} \Gamma ^0=&\begin{bmatrix} -I &{} {\mathcal {A}} &{} {\mathcal {B}} &{} {\mathcal {H}} &{} 0\\ 0 &{} {\mathcal {A}}_q &{} 0 &{} G &{} 0 \end{bmatrix}, \Delta ^0 = \begin{bmatrix} R_1^T &{} S_1^T &{} Z_1^T &{} U_1^T &{} V_1^T\\ R_2^T &{} S_2^T &{} Z_2^T &{} U_2^T &{} V_2^T \end{bmatrix}^T . \end{aligned}$$

One can check with an immediate calculation that (42) can be written as

43 $$\begin{aligned} \Omega ^0 =&\Gamma _{\bot }^T \Lambda \Gamma _{\bot } < 0, \end{aligned}$$

where $\Lambda$ is given in (33), and

$$\begin{aligned} \Gamma _{\bot }= \begin{bmatrix} I &{} 0 &{} -I &{} 0 &{} 0 &{} 0 &{} 0\\ 0 &{} I &{} {\mathcal {A}}^T &{} 0 &{} 0 &{} 0 &{} {\mathcal {A}}_q^T \\ 0 &{} 0 &{} {\mathcal {B}}^T &{} I &{} 0 &{} 0 &{} 0\\ 0 &{} 0 &{} {\mathcal {H}}^T &{} 0 &{} I &{} 0 &{} G^T\\ 0 &{} 0 &{} 0 &{} 0 &{} 0 &{} I &{} -I \end{bmatrix}^T. \end{aligned}$$

Then

$$\begin{aligned} \Gamma = \begin{bmatrix} -I &{} {\mathcal {A}} &{} -I &{} {\mathcal {B}} &{} {\mathcal {H}} &{} 0 &{} 0\\ 0 &{} {\mathcal {A}}_q &{} 0 &{} 0 &{} G &{} -I &{}-I \end{bmatrix}. \end{aligned}$$

With the application of Finsler’s lemma ii) and iii) it follows that $\Omega ^0<0$ is equivalent to the existence of a $\Delta$ given in (33) such that

44 $$\begin{aligned} \Lambda + \Delta \Gamma + \Gamma ^T \Delta <0. \end{aligned}$$

Set $\Gamma$ as the sum $\Gamma = \Gamma _1 + {\mathcal {I}} \tilde{\Gamma }_2,$ where $\Gamma _1$ is given by (34), and $\tilde{\Gamma }_2$ is defined by

$$\begin{aligned} \tilde{\Gamma }_2&= \begin{bmatrix} {\hat{H}}_1 &{} {\hat{A}}_{12} \\ {\hat{H}}_2 &{} {\hat{A}}_{22} \end{bmatrix} \left[ \begin{array}{ccccccccccccccccc} 0 &{} 0 &{} 0 &{} C_1 &{} C_2 &{} C_2 &{} 0 &{} 0 &{} 0 &{} B_y &{} 0 &{} H_y &{} 0 &{} 0 &{} 0 &{} 0 \\ 0 &{} 0 &{} 0 &{} 0 &{} 0 &{} I &{} 0 &{} 0 &{} 0 &{} 0 &{} 0 &{} 0 &{} 0 &{} 0 &{} 0 &{} 0 \end{array} \right] , \end{aligned}$$

then (44) can be rewritten in the form of

45 $$\begin{aligned} \Lambda + \Delta \Gamma _1 + \Gamma _1^T \Delta + \Delta {\mathcal {I}} \tilde{\Gamma }_2 + \tilde{\Gamma }_2^T {\mathcal {I}}^T \Delta ^T <0. \end{aligned}$$

Let *F* and $\Upsilon$ be matrices defined in the theorem. Add and subtract the matrix $\Upsilon F \tilde{\Gamma }_2$ and its transpose to the left hand side of inequality (45), and introduce the new variables ${\overline{A}}_{12}=F_1 {\hat{A}}_{12},$ ${\overline{A}}_{22}=F_2 {\hat{A}}_{22},$ ${\overline{H}}_{1}=F_1 {\hat{H}}_1$ ${\overline{H}}_{2}=F_2{\hat{H}}_1.$ Then one obtains that $\Gamma _2=F \tilde{\Gamma }_2,$ and (45) can equivalently be written as

46 $$\begin{aligned} \Lambda +\Delta \Gamma _{1}+ {\Gamma _{1}}^T \Delta ^T -\Upsilon \Gamma _{2}-{\Gamma _{2}}^T\Upsilon ^T +\left( \Delta {\mathcal {I}} + \Upsilon F \right) \tilde{\Gamma }_2 + \tilde{\Gamma }_2^T \left( \Delta {\mathcal {I}} + \Upsilon F \right) ^T&<0. \end{aligned}$$

The application of Lemma 2 verifies that inequalities (46) and (32) are equivalent, which completes the proof.

## Application to the estimation of the Hungarian potential GDP

In this section, it will be shown, how the robust filter design methods of the previous section can be applied to the models of Sect. 3. First, the data of Model 3 will be given in details.

The model variables of Sect. 3 and the variables of the general models of Sect. 4 can be identified as follows:

47 $$\begin{aligned} x(t)&=\begin{bmatrix} y_p(t) \\ g(t) \\ u_p(t) \\ u_c(t) \\ \pi _p(t) \\ \pi _c(t) \\ y_c(t) \\ y_c^{*}(t) \end{bmatrix}, w(t)= \begin{bmatrix} w^{(y_p ) } (t) \\ w^{(g ) } (t) \\ w^{(u_p ) } (t) \\ w^{(u_c) } (t) \\ w^{(\pi _p ) } (t) \\ w^{(\pi _c) } (t) \\ w^{(y_c ) } (t) \\ w^{(y_c^{*} ) } (t) \end{bmatrix}, y(t)= \begin{bmatrix} y(t) \\ u(t) \\ \pi (t) \end{bmatrix}, z(t)= \begin{bmatrix} y_p(t) \\ u_p(t) \\ \pi _p (t) \end{bmatrix}. \end{aligned}$$

(Emphasize that, in (47), — with a small abuse of notations - *y*(*t*) on the left hand side denotes the measured output variable of the general model, while it is the GDP on the right hand side!)

Then Model 3 can be given in matrix–vector notation form as

48 $$\begin{aligned} x(t+1)&=A_x x(t)+ H_{x}p_x(t)+B_x w(t), q_x(t) = A_{q} x(t), \end{aligned}$$

49 $$\begin{aligned} y(t)&=C_y x(t) , \end{aligned}$$

50 $$\begin{aligned} z(t)&= Lx(t). \end{aligned}$$

where $H_x \in {\textbf{R}}^{n_x\times 0}$ and $A_{q}\in {\textbf{R}}^{0\times n_x}$ (i.e. empty matrices), if model ${\mathcal {S}}_1$ is considered and they are given below for model ${\mathcal {S}}_2.$ Further,If system ${\mathcal {S}}_1$ is considered, then $a_1,$ $a_2$ are obtained as the convex combination of the four vertices $a_1^{ij}=\varrho _i \cos \omega _j,$ and $a_2^{ij}= \varrho _i \sin \omega _j,$ with given $(\varrho _i,\omega _j)$($i,j=1,2$), while $\gamma _u$ and $\gamma _{\pi }$ are taken to be fixed in order to avoid large computational burden.

If system ${\mathcal {S}}_2$ is considered, then $A_x$ is taken with fixed parameters $\gamma _u=\gamma _u^0,$ $\gamma _{\pi }=\gamma _{\pi }^0,$ $a_1=a_1^{0}=\varrho _0 \cos \omega _0,$ and $a_2=a_2^{0}= \varrho _0 \sin \omega _0,$ with given $(\varrho _0,\omega _0).$ Further, ${p_x}_1(t) = \varphi _u \left( t,{q_x}_1(t)\right) ,$ ${q_x}_1(t)= \bar{\delta }_1 x_7(t),$ and ${p_x}_2(t) = \varphi _{\pi } \left( t,{q_x}_2(t)\right) =\delta _2 (t) {q_x}_2(t),$ ${q_x}_2(t)= \bar{\delta }_2 x_7(t),$ ${p_x}_i(t) = {\delta }_i(t){q_x}_i(t),$ $${q_x}_i(t)= \bar{\delta }_i \begin{bmatrix} x_7(t)&x_8(t) \end{bmatrix}^T,$$ ($i=3,4$), where $\bar{\delta }_j$ ($j=1,\ldots ,4$) are given positive numbers and $|\delta _i(t)| \le 1,$ ($i=2,3,4$). Thus

51 $$\begin{aligned} A_q=\begin{bmatrix}0_{6\times 6}&\bar{\Delta } \end{bmatrix}, \text{ with } \bar{\Delta }=\begin{bmatrix}\bar{\delta }_1 &{} \bar{\delta }_2 &{} \bar{\delta }_3 &{} 0 &{} \bar{\delta }_4 &{} 0 \\ 0 &{} 0 &{} 0 &{} \bar{\delta }_3 &{} 0 &{} \bar{\delta }_4 \end{bmatrix}^T, \end{aligned}$$

and $$Q_{01}=-1,$$ $$S_{01}=b/2,$$ $$R_{01}=0,$$ $$Q_{02}=-1,$$ $$S_{02}=0,$$ $$R_{02}=1,$$ $$Q_{0i}=-I_2,$$ $$S_{0i}=0_2,$$ $$R_{0i}=I_2,$$ (i=3,4).

The data for Models 1 and 2 can be obtained by logically omitting variables, rows an columns in matrices given above.

The quarterly potential GDP of Hungary has been determined for Hungary by energy-to-peak robust filtering based on Theorem 1 and 2 for the period 2000–2021. Calculations were carried out with all the three models introduced in Sect. 3. The cycle has been modelled by dynamics (3). One can see that the relaxed stability condition required to apply the robust filter (see Sect. 3) has been satisfied. In all model runs, two different cycle lengths have been considered, when $\omega$ is around either $2\pi /80$ (the case of long cycle) or around $2\pi /30$ (the case of short cycle). (Similar cycle lengths have been considered in Bulligan et al. (2019) (30 and 85 quarters). Evans (2018) considered 36–144 months, the average of which corresponds to our shorter cycle length.) The numerical values of parameters are given in Table 1.

**Table 1:** Numerical data of parameters and adjustable variables

${\mathcal {S}}_1$	$\gamma _u=-0.02$	$\gamma _{\pi }= 0.01$					
	$\varrho _1= 0.96$	$\varrho _2=0.97$	$\omega _1=2\pi / 81$	$\omega _2=2\pi / 79$			Long cycle
	$\varrho _1=0.90$	$\varrho _2=0.92$	$\omega _1=2\pi / 33$	$\omega _2=2\pi / 30$			Short cycle
${\mathcal {S}}_2$	$\gamma _u^0=-0.01$	$\gamma _{\pi }^0= 0.01$	$\bar{\delta }_1=10\gamma _u^0$	$\bar{\delta }_2= 0.005$	$b=1/5$		
	$\varrho _0=0.965$	$\omega _0= 2\pi / 80$	$\bar{\delta }_3= 0.0052$	$\bar{\delta }_4= 0.0014$			Long cycle
	$\varrho _0=0.91$	$\omega _0=2\pi / 31$	$\bar{\delta }_3=0.0118$	$\bar{\delta }_4= 0.011$			Short cycle
	$$b_1=0.825 $$	$$ b_2=0.275$$	$$ b_3=0.125$$	$$ b_4=-0.085$$	$$b_5=0.15 $$	$$b_6=0.14886 $$	
	$$ d_1=0.7$$	$$ d_2=0.575$$	$$d_3=-0.3 $$	$$d_4=-0.25 $$	$$d_5=0.275 $$	$$d_6=0.275 $$	
	$${c_1}= 0.08 \ ^{\left. *\right) }$$	$$c_1=0.35 \ ^{\left. **\right) }$$	$$c_2=0.35 $$	$$c_3=0.35 $$		$$\beta = 0.2985$$	

$$^{\left. *\right) }$$ for $${\mathcal {S}}_1$$    $$^{\left. **\right) }$$ for$${\mathcal {S}}_2$$

With the data given in Table 1, system ${\mathcal {S}}_2$ allows a wither range of the parameters’ variations, then ${\mathcal {S}}_1.$

**Fig. 1:**
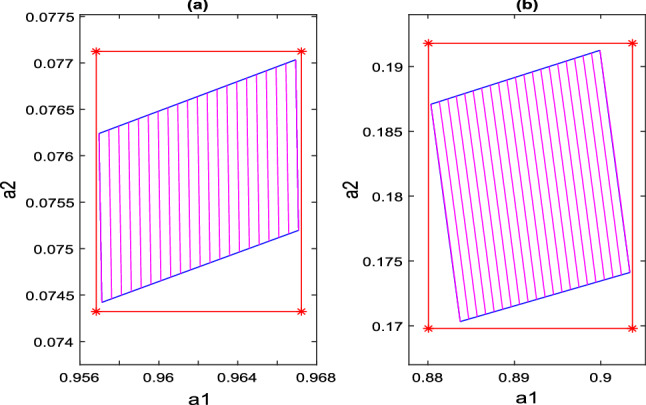
Uncertainty domains for $a_1$–$a_2$: **a** the case of long cycle, **b** the case of short cycle

On Fig. 1, the red rectangle and the polytope with blue boundary depict the allowable range of variations of the parameters $a_1$ and $a_2$ for systems ${\mathcal {S}}_2$ and ${\mathcal {S}}_1,$ respectively. The contribution of $y_c$ to the right hand sides of Eqs. (5) and (7) is $-0.03 y_c \le \gamma _u^0 y_c + \varphi _u \left( t,y_c\right) \le -0.01 y_c$ and $0.005 y_c \le \gamma _{\pi }^0 y_c \le 0.015 y_c$ in the case of ${\mathcal {S}}_2,$ while it is $-0.02 y_c$ and $0.01 y_c$ in the case of ${\mathcal {S}}_1$, respectively.

Consider now the results beginning with the simplest Model 1 and continuing with the more involved Model 2 and Model 3.

**Fig. 2:**
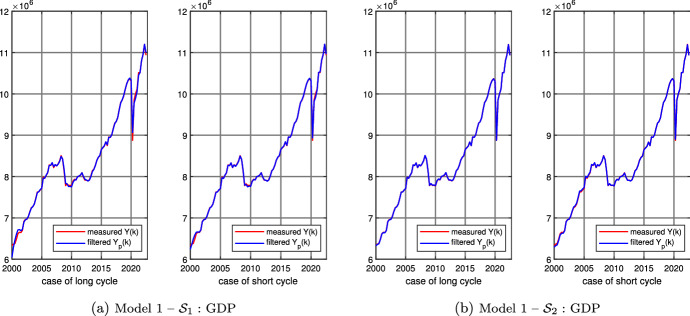
GDP results for Model 1 with polytopic (${\mathcal {S}}_1$) and quadratically bounded (${\mathcal {S}}_2$) uncertainties

*Model 1* First, consider the results of the univariate Model 1. The application of Theorem 1 resulted in the energy-to-peak performance bounds $\gamma =5.03$ for the long cycle case, and $\gamma =3.11$ for the short cycle case, while Theorem 2 yielded $\gamma =4.22$ for the long cycle case, and $\gamma =2.79$ for the short cycle case. Considering either system ${\mathcal {S}}_1$ or ${\mathcal {S}}_2$, the results are similar. The filtered and the actual values of the GDP are very close to each other (see Fig. 2a, b), although the filter could dampen the volatility, especially around the troughs of the two crises.

Even the multivariate models used to determine potential GDP are very simple in terms of systems paramteres. The successful application strongly influenced by their appropriate choice: the parameter values should be adjusted to the specifically examined economy. Furthermore, not only the specific parameter values may be different, but it cannot be expected that the same model would be equally successful for different national economies.

**Fig. 3:**
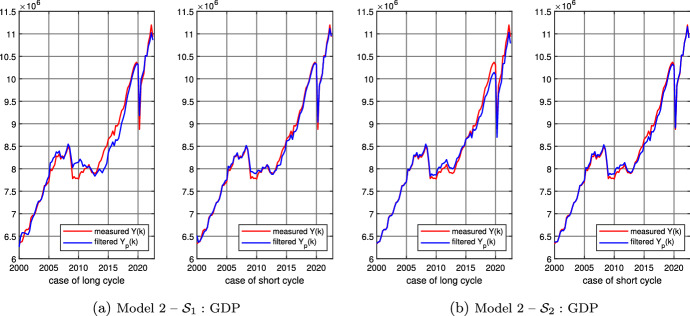
GDP results for Model 2 with polytopic (${\mathcal {S}}_1$) and quadratically bounded (${\mathcal {S}}_2$) uncertainties

**Fig. 4:**
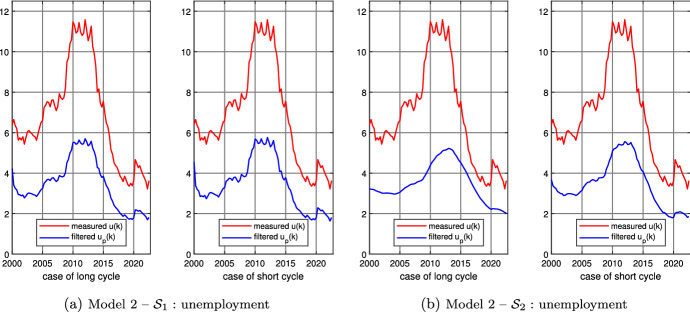
Unemployment results for Model 2 with polytopic (${\mathcal {S}}_1$) and quadratically bounded (${\mathcal {S}}_2$) uncertainties

*Model 2* In this case we filtered for both the GDP and the unemployment rate. The results for Model 2 are depicted on Fig. 4a, b. The application of Theorem 1 resulted in the energy-to-peak performance bounds $\gamma =5.03$ for the long cycle case, and $\gamma =2.69$ for the short cycle case, while Theorem 2 yielded $\gamma =4.24$ for the long cycle case, and $\gamma =2.79$ for the short cycle case. In the case of Hungary, the experiments with Model 2 proved to be relatively stable. One can observe on Fig. 3a, b that the dynamic character of the GDP and that of the filtered time-series are similar if either cycle length is chosen.

The application of long cycle shows a more characteristic result, as there are greater differences between the GDP and the filtered values. The financial world crisis affected the Hungarian economy until 2012, when the measured values were lower than the filtered ones. Since then, the Hungarian economic policy was procyclical until the Covid-19 crisis as Fig. 3a, b show, i.e., the GDP gap was positive. If system ${\mathcal {S}}_2$ was considered (with quadratically bounded uncertainty), one can see on Fig. 3a, b that just before the Covid-19 crisis, the GDP gap increased. In fact, the inflation rate increased, and the external balance deteriorated. After the Covid-19 crisis, the economy bounced back quickly, and the growth-focussed procyclical economic policy returned. Our calculations show that — in spite of the Covid-19-induced relapse — the GDP gap was still positive at the end of 2021. Figure 4a, b show that the impact of the financial crisis on unemployment rate was significantly higher and longer than that of the Covid-19. The measured unemployment became far higher than its natural rate as the consequence of the financial crisis, and the difference was far lower from 2015, when the GDP gap was positive. This is consistent with the calculated potential GDP. We remark that the estimated values of coefficients of the models depend on the actual economic policy measures of the examined economy. The dynamic equations of the model formulate relationships of the market economy, while the measures might go against or at least dampen the market reactions. This is also true for the $\gamma _u$ in Model 2 (and in Model 3 below, as well). For example, in Hungary the unemployment has been treated by an extensive public work program launched after 2010 that distorted the unemployment rate in certain years before the Covid-19 crisis. The benefit period is just three months in Hungary, which is exceptionally short in the European Union. After it these people may be employed as public — or fostered — workers earning well below the minimum wage for some low value-added work, but they are counted as employed in the statistics. This means that the difference between the natural and the actual rate of unemployment would be larger without such a measure.

**Fig. 5:**
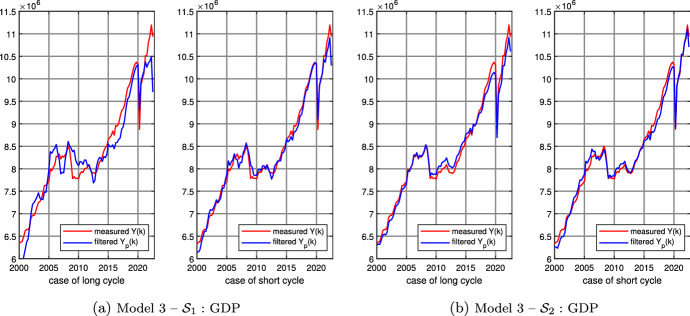
GDP results for Model 3 with polytopic (${\mathcal {S}}_1$) and quadratically bounded (${\mathcal {S}}_2$) uncertainties

**Fig. 6:**
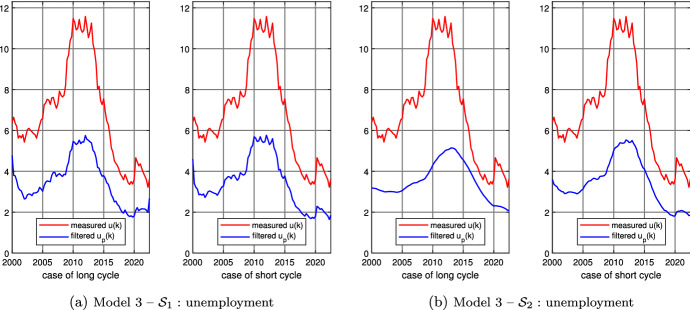
Unemployment results for Model 3 with polytopic (${\mathcal {S}}_1$) and quadratically bounded (${\mathcal {S}}_2$) uncertainties

**Fig. 7:**
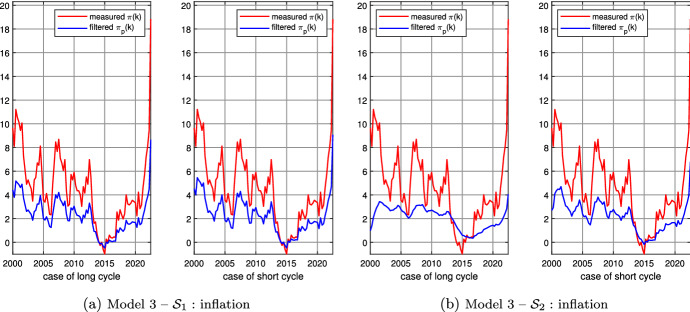
Inflation results for Model 3 with polytopic (${\mathcal {S}}_1$) and quadratically bounded (${\mathcal {S}}_2$) uncertainties

*Model 3* The results for Model 3 are depicted on Figs. 5a, 6 and 7b. The application of Theorem 1 resulted in the energy-to-peak performance bounds $\gamma =5.03$ for the long cycle case, and $\gamma =2.69$ for the short cycle case, while Theorem 2 yielded $\gamma =4.24$ for the long cycle case, and $\gamma =2.79$ for the short cycle case. The results of this model are consistent with those of Model 2 applying either type of uncertainty and either cycle length. Again, the filtering of ${\mathcal {S}}_2$ with long cycle shows the procyclical economy before and after the Covid-19 crisis (see Fig. 5b). The largest positive GDP gap can be observed in 2017–2019, when the inflation increased and the external balance began to deteriorate at a high rate of economic growth. The deep Covid-19 crisis did not the reduced the GDP gap for a longer time, while also the central budget deficit became very high.

The difference between the actual unemployment rate and its filter value was high during both crises, but in Model 3, the actual rates were almost equal to the filtered values in the second half of the 2010s because of the expansive economic policy. Corresponding to the procyclical, growth-focussed economic policy, the actual inflation was always higher than its reference level, except in 2015, when the rate was slightly below zero, see Fig. 7a, b. We remark however that Hungary is an extremely open economy. In this case the inclusion of the inflation rate as formulated in Model 3 might distort the results. Namely, in case of economic downturn the way of adjustment can be the weakening of the domestic currency, which may even raise the inflation, i.e. the expected positivity of $\gamma _\pi$ may not hold true for certain periods.

Since past values can only be computed, but not observed by statistics, there is no objective criterion for comparing the goodness of the results. All the experimental runs proved to be robust, model ${\mathcal {S}}_2$ with the longer cycle length provided the most characteristic results, which can be justified in economic terms, too.

## Conclusions

In this paper a new filter method is proposed, which is suitable to determine non-observable economic indicators, e.g. the potential GDP, which is a crucial information both for policymakers and for analysts. The main advantage of the proposed robust filter method over the traditional Kalman filter is that we do not have to make stochastic assumptions that may not be valid for the problem at hand. Instead, we can take into account the uncertainties of the dynamic system using unknown deterministic parameters and/or nonlinear functions. This means that a new model, not yet used in the literature, was applied to estimate the potential GDP. Uncertainties were taken into account in two ways. One is the application of a linear parameter varying (LPV) system, the other is the use of quadratically bounded uncertainties. In contrast to robust filtering results known from the literature, certain partial stability of the nominal system is sufficient for the proposed methods, which is a theoretically new result.

The proposed method was applied to estimate the Hungarian potential GDP based on quarterly data. We tested uni-, bi- and trivariate to estimate the Hungarian potential GDP in 2000–2021. Such estimation has not been published yet. The examined period included both the financial world crisis and the Covid-19 crisis. Similarly to several papers, two different cycle lengths have been taken into account. The results have been consistent, but the most characteristic results were provided by the bi- and trivariate models with quadratically bounded uncertainties with the longer cycle length. It turned out that the economic policy was very procyclical after 2012, and the GDP gap was still positive during and after the Covid-19 crisis.

## Appendix

For convenience of the readers, two lemmas needed for the proof of Theorem 2 is given below.

### Lemma 1

de Oliveira et al. (2001) (Finsler’ s lemma) Let $x \in {\textbf{R}}^{n},$ ${\mathcal {Q}}={\mathcal {Q}}^T \in {\textbf{R}}^{n \times n}$ and ${\mathcal {B}} \in {\textbf{R}}^{m \times n}$ such that $rank ({\mathcal {B}})<n.$ Then, the following statements are equivalent:

$$\begin{aligned} \mathrm{(i)} &x^T {\mathcal {Q}} x<0, \forall {\mathcal {B}}x=0, x \ne 0.\\ \mathrm{(ii)} &{\mathcal {B}}_{\bot }^T {\mathcal {Q}} {\mathcal {B}}_{\bot }<0, \text{ where } {\mathcal {B}}_{\bot } \text{ is } \text{ the } \text{ kernel } \text{ of } {\mathcal {B}}; \text{ i.e. } {\mathcal {B}}{\mathcal {B}}_{\bot } = 0; \\ \mathrm{(iii)} &\exists {\mathcal {X}} \in {\textbf{R}}^{n \times m}: \; {\mathcal {Q}}+{\mathcal {X}}{\mathcal {B}}+{\mathcal {B}}^T{\mathcal {X}}^T<0. \end{aligned}$$

### Lemma 2

Chang et al. (2015) For scalar $\beta$ and for matrices $\Sigma ,$ $\Xi ,$ $\Gamma ,$ *G* of compatible dimension the following statements are equivalent:

$$\begin{aligned} \mathrm{(i)} &\begin{bmatrix} \Sigma &{} * \\ \beta \Xi +G\Gamma &{} -\beta \left( G+G^T\right) \end{bmatrix}< 0, \\ \mathrm{(ii)} & \Sigma<0, \; \Sigma +\Xi \Gamma +\Gamma ^T\Xi ^T<0. \end{aligned}$$
